# Clinical and metabolic features of the randomised controlled Diabetes Remission Clinical Trial (DiRECT) cohort

**DOI:** 10.1007/s00125-017-4503-0

**Published:** 2017-11-30

**Authors:** Roy Taylor, Wilma S. Leslie, Alison C Barnes, Naomi Brosnahan, George Thom, Louise McCombie, Naveed Sattar, Paul Welsh, Carl Peters, Sviatlana Zhyzhneuskaya, Kieren G. Hollingsworth, Ahmad Al-Mrabeh, Angela M. Rodrigues, Lucia Rehackova, Ashley J. Adamson, Falko F. Sniehotta, John C. Mathers, Hazel M. Ross, Yvonne McIlvenna, Sharon Kean, Ian Ford, Alex McConnachie, Michael E. J. Lean

**Affiliations:** 1 Newcastle Magnetic Resonance Centre, Institute of Cellular Medicine, Campus for Ageing and Vitality, Newcastle University, Newcastle upon Tyne, UK; 2 Human Nutrition, School of Medicine, Dentistry and Nursing – GRI Campus, College of Medical, Veterinary and Life Sciences, University of Glasgow, 2nd Floor, New Lister Building, Glasgow Royal Infirmary, 10–16 Alexandra Parade, Glasgow, G31 2ER UK; 3 Human Nutrition Research Centre, Institute of Health and Society, Newcastle University, Newcastle upon Tyne, UK; 4 Counterweight Ltd, Corby, Northants UK; 5 Institute of Cardiovascular and Medical Science, University of Glasgow, Glasgow, UK; 6 Institute of Health and Society, Faculty of Medical Sciences, Newcastle University, Newcastle upon Tyne, UK; 7 Human Nutrition Research Centre, Institute of Cellular Medicine, Campus for Ageing and Vitality, Newcastle University, Newcastle upon Tyne, UK; 8 General Practice and Primary Care, Institute of Health and Wellbeing, University of Glasgow, Glasgow, UK; 9 Robertson Centre for Biostatistics, Institute of Health and Wellbeing, University of Glasgow, Glasgow, UK

**Keywords:** Formula diet, Remission, Type 2 diabetes, Weight management

## Abstract

**Aims/hypothesis:**

Substantial weight loss in type 2 diabetes can achieve a return to non-diabetic biochemical status, without the need for medication. The Diabetes Remission Clinical Trial (DiRECT), a cluster-randomised controlled trial, is testing a structured intervention designed to achieve and sustain this over 2 years in a primary care setting to determine practicability for routine clinical practice. This paper reports the characteristics of the baseline cohort.

**Methods:**

People with type 2 diabetes for <6 years with a BMI of 27–45 kg/m^2^ were recruited in 49 UK primary care practices, randomised to either best-practice diabetes care alone or with an additional evidence-based weight management programme (Counterweight-Plus). The co-primary outcomes, at 12 months, are weight loss ≥15 kg and diabetes remission (HbA_1c_ <48 mmol/mol [6.5%]) without glucose-lowering therapy for at least 2 months. Outcome assessors are blinded to group assignment.

**Results:**

Of 1510 people invited, 423 (28%) accepted; of whom, 306 (72%) were eligible at screening and gave informed consent. Seven participants were later found to have been randomised in error and one withdrew consent, leaving 298 (176 men, 122 women) who will form the intention to treat (ITT) population for analysis. Mean (SD) age was 54.4 (7.6) years, duration of diabetes 3.0 (1.7) years, BMI 34.6 (4.4) kg/m^2^ for all participants (34.2 (4.2) kg/m^2^ in men and 35.3 (4.6) kg/m^2^ in women) and baseline HbA_1c_ (on treatment) 59.3 (12.7) mmol/mol (7.6% [1.2%]). The recruitment rate in the intervention and control groups, and comparisons between the subgroups recruited in Scotland and England, showed few differences.

**Conclusions/interpretation:**

DiRECT has recruited a cohort of people with type 2 diabetes with characteristics similar to those seen in routine practice, indicating potential widespread applicability. Over 25% of the eligible population wished to participate in the study, including a high proportion of men, in line with the prevalence distribution of type 2 diabetes.

**Trial registration:**

www.controlled-trials.com/ISRCTN03267836; date of registration 20 December 2013

## Introduction

In the early years after diagnosis type 2 diabetes has been shown to be a reversible metabolic condition [1, 2]. Within 10 years of type 2 diabetes diagnosis, about two-thirds of individuals can return to non-diabetic levels of blood glucose control after diet-induced weight loss averaging 15 kg [3]. With stable reduced weight, non-diabetic blood glucose control and changes in the underlying pathophysiological abnormalities persist without glucose-lowering medications for at least 6 months [3]. However, these results have only been observed in a small number of motivated volunteers attending a specialist research unit. The practical implications for routine clinical practice of such diet-induced type 2 diabetes reversal urgently need to be established given widening public interest in this phenomenon.

Bariatric surgery of any kind can reverse metabolic abnormalities in about 70% of people with type 2 diabetes [4], with the likelihood of benefit determined primarily by the extent of weight loss and duration of diabetes [5, 6]. However, with conventional dietary advice mean weight loss is only 3–5% and remission is rare [7]. In the LookAHEAD study, a greater mean weight loss of 8% at 12 months produced remission of type 2 diabetes in 11.5% of participants [8]. The present study was designed to use a more intensive initial ‘total diet replacement’ approach, using a low-energy formula diet with a structured behaviour change programme for long-term weight loss maintenance, delivered in a realistic primary care setting. In a feasibility study, this programme resulted in a mean weight loss of about 17 kg by 12 weeks, with 40% remaining >10 kg and 33% >15 kg below baseline weight at 12 months [9]. Similar figures have been reported for a roll-out of the Counterweight-Plus programme to 288 individuals in routine primary care (L. McCombie, N. Brosnahan, H. Ross, A. Bell-Higgs, L. Govan, M. E. J. Lean, unpublished results). The Diabetes Remission Clinical Trial (www.controlled-trials.com/ISRCTN03267836) was designed to answer a series of linked questions [10]: What proportion of the whole population with type 2 diabetes would agree to undertake substantial weight loss? How many of these could achieve ≥15 kg weight loss and return to non-diabetic blood glucose control off medication (the twin primary endpoints) at 1 year? What happens to the major pathophysiological factors underlying type 2 diabetes during continued follow up? What are the psychological consequences of the programme and can outcomes be predicted by psychometric variables? What proportion of the intervention group remains non-diabetic after 2 years? This paper describes the recruitment process, outcomes of recruitment and baseline characteristics of the study participants.

## Methods

A cluster-randomised controlled design was employed, with general practices the unit of randomisation. General practices representing populations with wide ranges of social and geographical features across Scotland and in the Tyneside region of northeast England were invited to participate by the Primary Care Research Network (PCRN) in Scotland and North East Commissioning Support (NECS) in England. Practices known to be research-active were approached first as the demands of research work by staff would be too great for many practices.

The research team met with interested practices to discuss participation details. Following these meetings, willing practices were then randomised to intervention or control. Practices randomised to the control group continued to deliver usual diabetes and weight management as per current clinical guidelines. Practices randomised to the intervention group continued to deliver usual guideline-based care in addition to an evidence-based weight management programme (Counterweight-Plus). Randomisation was conducted independently by the Robertson Centre for Biostatistics, University of Glasgow, using a minimisation method to maintain balance across groups between the two study regions (Scotland and Tyneside), including a balance of practice list sizes (*n* > 5700 or *n* ≤ 5700).

Within each general practice, all people with type 2 diabetes meeting inclusion and exclusion criteria were identified by a computerised search of general practitioner (GP) records, undertaken by PCRN in Scotland and GP practice staff in Tyneside. Lists generated by the search were reviewed by GPs and any individuals recognised to be unsuitable to approach because of comorbidity or other practical obstacles to participation were removed.

Inclusion criteria were age 20–65 years, type 2 diabetes of duration 0–6 years (diagnosis based on two recorded diagnostic tests using blood glucose and/or HbA_1c_ [HbA_1c_ >48 mmol/mol (6.5%) on diet alone or HbA_1c_ >43 mmol/mol (6.1%) on treatment with oral glucose-lowering agents]), and a BMI of 27–45 kg/m^2^. Potentially eligible individuals were mailed an invitation to participate with an information sheet and asked to respond using prepaid reply envelopes. Full details of methodology have been previously described [10]. A subsequent protocol amendment included the provision of a financial incentive (50 GBP Amazon retail voucher) for those recruited into the control arm. The intervention arm already provided a desirable incentive ‘in kind’ for participation (supply of a formula diet and potential substantial weight loss) but there had been no such incentive for participants in control practices.

To ascertain whether the individuals volunteering and included were representative of the wider population with type 2 diabetes, defined by the inclusion and exclusion criteria, anonymised data on sex, height and weight, duration of diabetes and index of multiple deprivation were collated for all who were invited to participate.

Ethics approval for the Diabetes Remission Clinical Trial (DiRECT) was secured on 24 January 2014. NHS research and development approvals for participating Health Board areas in Scotland and Clinical Commissioning Groups in Tyneside were then secured. All subsequent protocol amendments have been similarly approved. Written informed consent was secured from all participants.

### Statistical analyses

Summary data are presented overall and by study area (Scotland, Tyneside). Continuous data are summarised by the mean and SD, and categorical data by frequencies and percentages. Study areas are compared using Wilcoxon–Mann–Whitney tests for continuous data and Fisher’s exact tests for categorical data. Summary data are presented to enable comparison of the DiRECT study population with the wider Scottish population and with other studies of type 2 diabetes in the USA, Canada and Europe. Summary data are also reported for all individuals who were invited to take part in DiRECT and for whom anonymous baseline data could be obtained for comparison with the recruited study population. At this stage of analysis of baseline data, the codes have not been broken to reveal which participants are in which treatment arm and investigators remain blinded. Baseline data for the treatment arms will be analysed later, together with the primary outcome data.

## Results

### Primary care practice recruitment

**Fig. 1:**
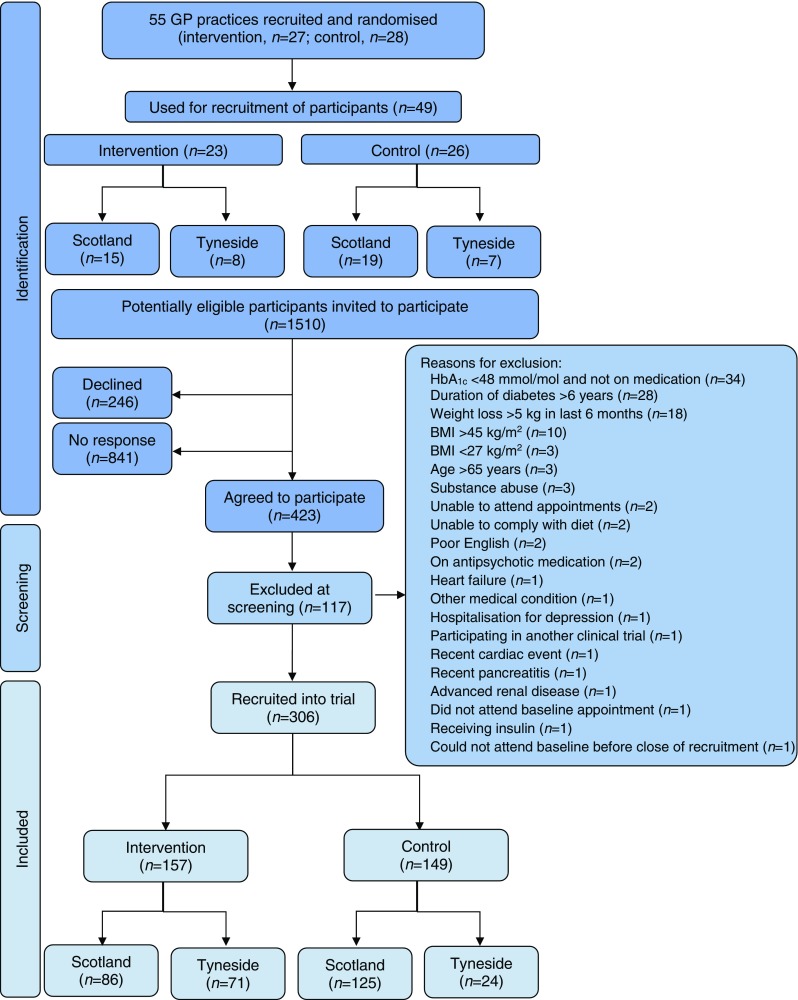
Flowchart of recruitment

Of the 523 practices that received an invitation to participate in the study, 74 expressed interest in participating. Following meetings with the study team, 55 practices agreed to participate and were randomised (34 Scotland, 21 Tyneside). The main factor for non-participation was the potential time commitment required if allocated to the intervention arm. Four practices allocated to intervention and two to control were not required for recruitment. Therefore, 49 practices were asked to recruit participants (Fig. 1), with list sizes varying between 1400 and 30,000 individuals in both urban and rural areas.

### Participant recruitment

A total of 1510 potentially eligible people were invited to participate in the study (Fig. 1). Of these, 423 (28%) accepted the invitation. The response was similar in the control and intervention arms (Table 1). Of those who wished to participate, 28% were excluded at screening. The most frequent reasons for exclusion were HbA_1c_ <48 mmol/mol (6.5%) without glucose-lowering medication, duration of type 2 diabetes >6 years and recent weight loss >5 kg (Fig. 1). From 48 practices, 306 participants gave consent and were recruited into the trial (no participants were recruited at one of the practices). After the baseline visit, one participant withdrew consent and seven participants were subsequently considered to have been randomised in error because their most recent HbA_1c_ was already in the non-diabetic range without drug treatment. These eight participants will not be included in the intention to treat (ITT) population for data analyses, and data are presented for the remaining 298 participants.

**Table 1:** Recruitment response by treatment allocation and study area

	Invited (*n*)	Accepted (*n*)	Response (%)
Intervention arm			
Scotland	464	130	28
Tyneside	315	85	27
Total	779	215	28
Control arm			
Scotland	552	166	30
Tyneside	179	42	23
Total	731	208	28
Total	1510	423	28

### Baseline characteristics

The baseline characteristics of the study population, detailed in Table 2, were similar for participants in Scotland and Tyneside. Mean age at recruitment was 54.4 years. Over half of the study population were male (59%). Mean BMI was slightly higher in women (35.3 kg/m^2^) than in men (34.2 kg/m^2^). Mean (SD) HbA_1c_ was 59.3 (12.7) mmol/mol (7.6% [1.2%]). Mean duration since diagnosis of type 2 diabetes was 3 years. Reflecting the ethnic composition of Scotland and Tyneside, 98% of participants were white, with 0.7% Black African, 0.6% South Asian and 0.3% other. The majority of participants were prescribed glucose-lowering medications (*n* = 226, 76%), most as monotherapy (*n* = 144, 48%), with metformin most commonly prescribed (*n* = 211, 71%). About a quarter of participants were managed by diet alone, and 82 (28%) were prescribed two or more glucose-lowering medications, including 63 (21%) receiving sulfonylureas. Few participants (12%) were current cigarette smokers and 50% reported never having smoked. Only 26% of participants reported any current or recent weight management activities. Table 3 shows the documented medical history of participants at baseline. Twelve per cent of participants had existing retinopathy. More than half of the study population had a history of hypertension and 55% of participants were prescribed antihypertensive medication, with over 30% on two or more drugs. The overall prevalence of cardiovascular disease (CVD; myocardial infarction, angina, stroke, transient ischaemic attack, heart failure, peripheral vascular disease, revascularisation, atrial fibrillation) was 12%. Forty three per cent of participants were prescribed six or more drugs and 13% ten or more. The proportion being treated with antidepressant drugs was 23%. Biochemical data at baseline (Table 4) were as expected among unselected individuals, predominantly with obesity and within 6 years of type 2 diabetes diagnosis.

**Table 2:** Baseline characteristics by study area (ITT population)

	All	Scotland	Tyneside	*p* value
*N*	298	197	101	
Sex, *n* (%)				
Male	176 (59.1)	118 (59.9)	58 (57.4)	0.710
Female	122 (40.9)	79 (40.1)	43 (42.6)	
Age (years)	54.4 (7.6)	55.2 (7.2)	52.8 (8.1)	0.014
Height (cm)				
Men	175.9 (7.1)	175.8 (7.0)	176.2 (7.2)	0.711
Women	160.7 (5.2)	160.6 (5.4)	160.8 (4.9)	0.684
Weight (kg)				
Men	106.0 (15.8)	105.7 (15.5)	106.5 (16.6)	0.704
Women	91.2 (13.0)	90.8 (13.9)	92.0 (11.4)	0.476
BMI (kg/m^2^)				
All	34.6 (4.4)	34.6 (4.4)	34.8 (4.4)	0.656
Men	34.2 (4.2)	34.2 (4.2)	34.2 (4.4)	0.929
Women	35.3 (4.6)	35.1 (4.8)	35.6 (4.4)	0.548
Waist circumference (cm)^a^				
Men	110.0 (7.1)	110.7 (6.2)	108.6 (8.7)	0.148
Women	101.6 (8.6)	101.7 (9.3)	101.4 (7.1)	0.831
Hip circumference (cm)^a^				
Men	108.6 (5.6)	109.4 (5.6)	107.1 (5.3)	0.066
Women	111.6 (8.0)	111.9 (8.4)	111.0 (7.1)	0.804
Systolic blood pressure (mm/Hg)	134.9 (16.9)	136.8 (16.4)	131.3 (17.2)	0.004
Diastolic blood pressure (mm/Hg)	85.0 (9.5)	85.5 (9.1)	84.1 (10.2)	0.088
Duration of type 2 diabetes (years)	3.0 (1.7)	3.0 (1.7)	2.9 (1.7)	0.710
Pre-trial HbA_1c_ (from GP records)				
mmol/mol	61.6 (13.9)	61.4 (14.2)	62.0 (13.4)	0.441
%	7.79 (1.27)	7.76 (1.30)	7.83 (1.23)	
Trial measured HbA_1c_				
mmol/mol	59.3 (12.7)	59.6 (13.4)	58.6 (11.2)	0.880
%	7.57 (1.16)	7.61 (1.22)	7.51 (1.03)	
Management of type 2 diabetes, *n* (%)				
Diet alone (no drugs)	72 (24.2)	38 (19.3)	34 (33.7)	0.018
1 drug	144 (48.3)	104 (52.8)	40 (39.6)	
≥2 drugs	82 (27.5)	55 (27.9)	27 (26.7)	
Smoking status, *n* (%)				
Current	35 (11.7)	21 (10.7)	14 (13.9)	0.663
Former	113 (37.9)	77 (39.1)	36 (35.6)	
Never	150 (50.3)	99 (50.3)	51 (50.5)	
Current/recent engagement in weight management activities, *n* (%)	77 (25.8)	62 (31.5)	15 (14.9)	0.002
Reported weekly alcohol intake (units)	5.5 (9.6)	5.5 (10.3)	5.7 (8.0)	0.040

Data are mean (SD) unless otherwise stated

^a^Waist and hip circumference was only measured when BMI was <35 kg/m^2^

**Table 3:** Medical history documented at baseline by study area (ITT population)

	All	Scotland	Tyneside	*p* value
*N*	298	197	101	
Diabetic retinopathy	35 (11.7)	20 (10.2)	15 (14.9)	0.256
Hypertension^a^	169 (56.7)	113 (57.4)	56 (55.4)	0.805
Current antihypertensive medication	163 (54.7)	113 (57.4)	50 (49.5)	0.220
1 drug	69 (23.2)	45 (22.8)	24 (23.8)	0.279
≥2 drugs	94 (31.5)	68 (34.5)	26 (25.7)	
Current antidepressant medication	68 (22.8)	44 (22.3)	24 (23.8)	0.773
Total number of prescribed medications				
None	6 (2.0)	3 (1.5)	3 (3.0)	0.280
1–2	47 (15.8)	30 (15.2)	17 (16.8)	
3–5	116 (38.9)	73 (37.1)	43 (42.6)	
6–9	89 (29.9)	59 (29.9)	30 (29.7)	
≥10	40 (13.4)	32 (16.2)	8 (7.9)	
Clinical CVD				
Myocardial infarction	8 (2.7)	7 (3.6)	1 (1.0)	0.273
Angina	8 (2.7)	6 (3.0)	2 (2.0)	0.721
Stroke	7 (2.3)	5 (2.5)	2 (2.0)	1.000
Transient ischaemic attack	7 (2.3)	7 (3.6)	0 (0.0)	0.100
Heart failure	2 (0.7)	2 (1.0)	0 (0.0)	0.550
Peripheral vascular disease	7 (2.3)	5 (2.5)	2 (2.0)	1.000
Previous revascularisation	4 (1.3)	4 (2.0)	0 (0.0)	0.304
Atrial fibrillation	8 (2.7)	7 (3.6)	1 (1.0)	0.273
Any CVD	37 (12.4)	30 (15.2)	7 (6.9)	0.042
Hyperlipidaemia^b^	67 (22.5)	48 (24.4)	19 (18.8)	0.307
eGFR <60 ml min^−1^ l.73 m^−2 c^	9 (3.1)	7 (3.6)	2 (2.1)	0.723
Arthritis	58 (19.5)	39 (19.8)	19 (18.8)	0.878
Osteoporosis	2 (0.7)	2 (1.0)	0 (0.0)	0.550
Epilepsy	2 (0.7)	1 (0.5)	1 (1.0)	1.000
Other	237 (79.5)	147 (74.6)	90 (89.1)	0.004

Data presented as *n* (%)

^a^Hypertension is defined as in the National Institute for Health and Care Excellence (NICE) guidelines for type 2 diabetes [25]

^b^Hyperlipidaemia is defined as in the NICE guidelines for cardiovascular disease [26]

^c^For eGFR, *n* = 196 for Scotland, *n* = 95 for Tyneside and *n* = 291 for Au (missing data are owing to missing creatinine values at baseline)

**Table 4:** Baseline biochemistry by study area (ITT population)

	All	Scotland	Tyneside	*p* value
*n* (missing)	291 (7)	196 (1)	95 (6)	
Total cholesterol (mmol/l)	4.32 (1.19)	4.38 (1.21)	4.19 (1.13)	0.239
HDL-cholesterol (mmol/l)	1.12 (0.28)	1.14 (0.28)	1.08 (0.29)	0.043
Triacylglycerol (mmol/l)	2.00 (1.17)	2.10 (1.27)	1.80 (0.89)	0.034
ALT (U/l)	34.1 (19.4)	34.8 (20.1)	32.7 (17.8)	0.450
AST (U/l)	24.2 (12.8)	24.8 (13.1)	22.8 (12.2)	0.136
GGT (U/l)	52.4 (56.8)	54.5 (62.8)	48.0 (41.7)	0.567
Creatinine (μmol/l)	68.3 (15.1)	70.7 (15.1)	63.3 (13.9)	<0.001
eGFR (ml min^−1^ 1.73 m^−2^)	98.6 (24.7)	94.3 (22.0)	107.5 (27.4)	<0.001
*n* (missing)	292 (6)	196 (1)	96 (5)	
Glucose (mmol/l)	9.01 (2.94)	9.30 (2.94)	8.43 (2.85)	0.010
Insulin (mU/ml)	23.50 (14.35)	25.34 (14.58)	19.67 (13.13)	<0.001
*n* (missing)	292 (6)	197 (0)	95 (6)	
ACR (mg/mmol)^a^	2.16 (6.89)	1.71 (6.73)	3.08 (7.16)	0.414
Microalbuminuria, *n* (%)^b^	39 (13.4)	18 (9.1)	21 (22.1)	0.003

Data are mean (SD) unless otherwise stated

^a^Values <0.5 imputed as 0.25

^b^Defined as ACR ≥3.5 (female) or ACR ≥2.5 (male)

## Discussion

DiRECT has a realistic or ‘pragmatic’ design to provide information about the management of type 2 diabetes in a routine primary care setting in a largely unselected population in order to maximise transferability of results. Certain inclusion criteria were applied for recruitment into DiRECT for various practical reasons. An upper BMI limit of 45 kg/m^2^ was set because the protocol required a subsample to undergo abdominal magnetic resonance imaging. An upper age limit of 65 years was fixed to avoid the greater mortality rates associated with older people in a study planned to continue for 2 years and to optimise attendance at study visits given the greater mobility problems often faced by older people with type 2 diabetes. Furthermore, individuals were excluded if they had suffered myocardial infarction or stroke within the 6 months prior to recruitment because of engagement in other programmes.

Volunteers for clinical research are usually relatively more health conscious and there can be a risk of attracting unrepresentative ‘concerned but healthy’ individuals. The DiRECT cohort included, at baseline, 12% with existing retinopathy, 12% with one or more known manifestations of heart disease and 13% with microalbuminuria indicating nephropathy. Impaired renal function (eGFR <60) was present in 3%. In addition to their diabetes, as may be expected, a high proportion (57%) had known hypertension and 31% were prescribed two or more antihypertensive medications. Polypharmacy was common, with 43% being prescribed six or more different drugs daily and 13% prescribed ten or more drugs. Almost a quarter of the entire cohort was prescribed antidepressant medications, some of which are associated with weight gain [11]. Thus, the DiRECT cohort does not appear to include an excess of ‘worried well’ or unusually healthy people with type 2 diabetes.

DiRECT recruited a substantial proportion of men. This is encouraging as this is in line with the higher prevalence of type 2 diabetes in men [12]. Comparison of the study population with available data for those invited to participate in DiRECT shows an almost identical proportion of men and women accepting the invitation to participate (Table 5). Typically, men are less likely to engage in weight management programmes [13–15]. However, the potential for health improvement is a motivator for weight loss in men [16, 17], so a potential remission of diabetes or improvement in diabetes status may have encouraged men to participate in DiRECT. Whether a total diet replacement approach might be more attractive to men than to women, and other motivational issues, will be explored in interviews with participants and with healthcare professionals involved in DiRECT.

**Table 5:** Comparison of invited participants with the DiRECT study population

	Invited population	DiRECT participants
*n*	1155^a^	298
Sex, *n* (%)		
Male	699 (61.3)	176 (59.1)
Female	442 (38.7)	122 (40.9)
Year of birth	1961 (8)	1961 (8)
BMI (kg/m^2^)	33.5 (6.9)	34.6 (4.4)
Duration of type 2 diabetes (years)	3.5 (3.2)	3.0 (1.7)
Index of Multiple Deprivation quintile, *n* (%)		
Q1 – Most deprived	257 (22.8)	63 (21.4)
Q2	185 (16.4)	52 (17.6)
Q3	226 (20.0)	64 (21.7)
Q4	238 (21.1)	67 (22.7)
Q5 – Least deprived	222 (19.7)	49 (16.6)

Data are mean (SD) unless otherwise stated

^a^Data available for 1141 invited participants. Missing data for each item: sex, *n* = 14 (invited); year of birth, *n* = 10 (invited); BMI, *n* = 22 (invited); duration of diabetes, *n* = 603 (invited); deprivation, *n* = 30 (invited = 27; DiRECT = 3)

Recruitment of participants to any study is challenging. Poor recruitment can result in inadequate study numbers and underpowered trials [18]. DiRECT was based in primary care in order to provide realistic results transferable to routine practice. Of the invited practices, 14% accepted, reflecting the relatively small proportion of general practices in the UK that are research-active. Many of those are at capacity for taking on new research projects, being busy NHS general practices with limited availability of doctor and nurse time for research. There was great interest in DiRECT, but many of the research-active practices had already committed to research studies on other topics. Moreover, practice participation required practices to be able to provide six to ten individuals who would meet the inclusion criteria; this excluded some smaller practices. Recruitment to DiRECT was monitored closely and recruitment methods were refined to include strategies known to maximise recruitment (e.g. reminder letter, telephone contact and incentive) [18]. It is remarkable that 28% of all invited to participate expressed interest in doing so, and most of those who initially volunteered participated (72%). This far exceeds many other trials that recruited using similar methods [19] and those that recruited by other means [20, 21]. The study recruited more than the numbers required to satisfy the a priori power calculation (140 per group) within the 2 years allocated for recruitment, and recruitment proved difficult to halt resulting in modest over-recruitment. The ready acceptance of a low-energy liquid diet has been observed in all of our previous studies [1, 3, 9].

Triacylglycerol, insulin, fasting glucose and eGFR were less good in Scottish participants, in keeping with the slightly greater age and higher waist circumferences. None of these differences could be considered clinically important. Average alanine aminotransferase (ALT) and γ-glutamyl transferase (GGT) levels were higher than the normal reference range. There were a small number with ALT and GGT raised more so than aspartate aminotransferase (AST). This will be partly the result of fatty liver, which is common in this participant group, but more substantial elevations in GGT without comparable ALT elevations suggest that some participants may have had undisclosed alcohol excess. Of the 12 participants with GGT >150 at baseline, only one reported very high alcohol consumption (72 units per week). In most instances transaminases were also elevated but there were no other features in the baseline data which might account for abnormal liver function tests. There were minor excesses of high albumin/creatinine ratio (ACR) in Tyneside, the reasons for which were not clear although the numbers are small and do not allow determination of whether such minor albuminuria was sustained or transient.

The participants can be considered representative of the general population of people with short duration type 2 diabetes, and their characteristics were similar to those of other large studies of type 2 diabetes (Table 6). Comparison with LookAHEAD [20] showed similar mean HbA_1c_ (55 mmol/mol [7.2%]) and BMI (35.9 kg/m^2^), low prevalence of CVD (14.1%) and a high proportion of participants never having smoked (50.2%), although the proportion of male participants was lower (40%) and the population slightly older. The prevalence of CVD was higher in the ACCORD [21] and PROactive [22] studies, but this was a specific inclusion criterion for both those studies. As in DiRECT, both of these studies had high proportions of male participants and a similar mean BMI. Median HbA_1c_ was higher in ACCORD and PROactive (64 mmol/mol [8%]). Importantly, duration of diabetes was longer in all three of these previous studies, while it was specifically limited to <6 years in the present study. This duration was selected in view of the demonstration of duration of type 2 diabetes being a critical factor in achieving remission [3]. Our study population was, however, very representative of all people with type 2 diabetes in Scotland, in whom the mean duration of diabetes was 9 years [23]. A greater number of medications were prescribed to the Scottish than to the Tyneside participants, perhaps reflecting variations in the application of the different clinical guidelines in Scotland [24] and England [25].

**Table 6:** Comparison of DiRECT participant characteristics with those of the wider population of people with type 2 diabetes in Scotland and with those recruited into other type 2 diabetes studies

	DiRECT	Scottish population of people with type 2 diabetes (2015/2016) [23]	LookAHEAD [20] (USA)	ACCORD [21] (USA/Canada)	PROactive [22] (Europe)
*N*	298	263,843	5145	10,251	5238
Sex (%)					
Male	59.1	57.5	40.6	61.3	66.5
Female	40.9	42.5	59.4	38.7	33.5
Mean age (years)	54.4	66.7	59.0	62.2	61.8
Mean BMI (kg/m^2^)	34.6	32.0	35.9	32.2	31.0
Duration of type 2 diabetes (years)	3.0	9.3	7.0	10	8
Mean HbA_1c_					
mmol/mol	59.3	59.1	56.0	67.0	63.0
%	7.6	7.6	7.3	8.3	7.9
History of hypertension (%)	56.7	n/a	80.3	n/a	75.6
Antihypertensive medication (%)	54.7	n/a	68.4	85.5	n/a
Smoking status (%)					
Current	11.7	n/a	4.4	14.0	13.5
Former	37.9		45.4	44.0	45
Never	50.3		50.2	42.0	n/a
History of CVD (%)	12.4	n/a	14.1	35.0	n/a

A remarkably high proportion of eligible people with type 2 diabetes volunteered to enter DiRECT, including more men than is usual for weight management trials. The participants recruited were broadly representative of the unselected general population of people with up to 6 years’ duration of type 2 diabetes, and the subset in Tyneside were similar to the Scottish subset. The recruitment rates into the intervention and control arms were almost identical. The results of the weight management intervention in DiRECT are thus likely to be widely applicable to people with type 2 diabetes.

## Abbreviations

ALT: Alanine aminotransferase
AST: Aspartate aminotransferase
CVD: Cardiovascular disease
DiRECT: Diabetes Remission Clinical Trial
GGT: γ-Glutamyl transferase
GP: General practitioner
ITT: Intention to treat
NECS: North East Commissioning Support
PCRN: Primary Care Research Network
